# Anti-TIM-4 Monoclonal Antibody Treatment Reduces Autoreactive CD8^+^ T Cells and Restores Euglycemia in New-Onset Diabetic NOD Mice

**DOI:** 10.3390/biom16101498

**Published:** 2026-10-14

**Authors:** Yoon-Ju Kim, You Hyun Jeon, Se-Hyun Oh, Yu-Kyung Chung, Hee-Yeon Jung, Ji-Young Choi, Jang-Hee Cho, Sun-Hee Park, Chan-Duck Kim

**Affiliations:** 1 Division of Nephrology, Department of Internal Medicine, School of Medicine, Kyungpook National University Hospital, Kyungpook National University, Daegu 41944, Republic of Korea; 2 Cell and Matrix Research Institute, Kyungpook National University, Daegu 41944, Republic of Korea

**Keywords:** TIM-4, type 1 diabetes, NOD mice, autoreactive CD8^+^ T cells

## Abstract

Type 1 diabetes (T1D) is driven by immune-mediated destruction and dysfunction of pancreatic β-cells, with autoreactive CD8^+^ T cells serving as important effectors of β-cell injury. T-cell immunoglobulin and mucin domain-containing protein 4 (TIM-4) is expressed predominantly by macrophages and subsets of antigen-presenting cells and participates in phosphatidylserine recognition, efferocytosis, and regulation of adaptive immune responses. We investigated whether short-course TIM-4 targeting alters diabetogenic antigen-specific CD8^+^ T-cell responses in female non-obese diabetic (NOD) mice with new-onset diabetes. Mice received an anti-TIM-4 monoclonal antibody (3 mg/kg, intraperitoneally, every 2 days for 4 doses). Autoreactive CD8^+^ T cells were quantified using H-2K^d^ tetramers loaded with an insulin peptide or the islet-specific glucose-6-phosphatase catalytic subunit-related protein (IGRP)_206–214_ peptide, and pancreatic histology and intracellular interferon-γ (IFN-γ), interleukin-17 (IL-17), and forkhead box P3 (FoxP3) staining were assessed. Euglycemia was restored in 8 of 11 treated mice (72.7%) despite cessation of therapy. Anti-TIM-4 monoclonal antibody treatment markedly reduced IGRP_206–214_-reactive CD8^+^ T-cell frequencies in pancreatic lymph nodes and pancreas compared with untreated diabetic mice and was associated with preservation of pancreatic islet architecture. Treatment was also associated with lower IFN-γ and IL-17 expression and a higher frequency of FoxP3^+^ CD4^+^ T cells in pancreatic lymph nodes. These findings identify TIM-4-associated immune pathways as potential modulators of diabetogenic antigen-specific CD8^+^ T-cell responses and support further mechanistic evaluation of TIM-4 targeting in autoimmune diabetes.

## 1. Introduction

Type 1 diabetes (T1D) is an immune-mediated disease characterized by the progressive loss and dysfunction of insulin-producing pancreatic β-cells. Both innate and adaptive immune mechanisms contribute to disease development, with autoreactive CD4^+^ and CD8^+^ T cells playing central roles in β-cell-directed autoimmunity in humans and in the non-obese diabetic (NOD) mouse model [1,2,3]. Interactions among β-cells, antigen-presenting cells (APCs), and autoreactive lymphocytes promote the activation and recruitment of pathogenic T cells, ultimately leading to progressive β-cell injury and loss of functional β-cell mass [1,2,3].

The NOD mouse develops spontaneous autoimmune diabetes and has been widely used to investigate disease mechanisms and potential immunotherapeutic strategies. Numerous interventions targeting T cells, inflammatory pathways, APCs, or β-cell stress can prevent or delay diabetes when administered before the onset of overt hyperglycemia. In contrast, reversal of established diabetes is considerably more difficult to achieve because substantial β-cell injury has already occurred, and pathogenic autoimmune responses are firmly established [4,5,6]. Nevertheless, several previous studies have demonstrated that combining immunomodulatory therapy with α1-antitrypsin treatment could successfully restore euglycemia in NOD mice with new-onset diabetes [7,8]. These findings suggest that recent-onset diabetes may retain a therapeutic window during which suppression of pathogenic inflammation and preservation of residual β-cell function can restore glycemic control.

Autoreactive CD8^+^ T cells are important direct effectors of β-cell injury. Recognition of β-cell-derived peptides presented by major histocompatibility complex (MHC) class I molecules promotes the activation and accumulation of antigen-specific CD8^+^ T cells within pancreatic islets [9,10,11]. Insulin and islet-specific glucose-6-phosphatase catalytic subunit-related protein (IGRP) are well-characterized targets of diabetogenic CD8^+^ T cells in NOD mice. In particular, CD8^+^ T cells recognizing the H-2K^d^-restricted IGRP_206–214_ epitope represent a well-characterized diabetogenic population associated with the progression of islet autoimmunity [9,10,11]. MHC class I tetramers containing defined β-cell-derived peptides therefore provide a useful approach for directly identifying and quantifying these autoreactive CD8^+^ T-cell populations.

T-cell immunoglobulin and mucin domain-containing protein 4 (TIM-4) is an immune-regulatory molecule expressed predominantly by macrophages and subsets of APCs. TIM-4 functions as a receptor for phosphatidylserine exposed on apoptotic cells and participates in their recognition and clearance through efferocytosis [12,13]. Beyond apoptotic cell clearance, TIM-4 can influence adaptive immune responses, including the expansion, persistence, and removal of antigen-specific T-cell populations [12,13,14]. Because persistent β-cell injury and autoantigen presentation sustain pathogenic T-cell responses in T1D, TIM-4-dependent pathways may influence the survival, activation, or tissue accumulation of diabetogenic T cells. However, the role of TIM-4 in established autoimmune diabetes, particularly in regulating β-cell antigen-specific CD8^+^ T-cell responses, remains incompletely understood.

We therefore investigated whether targeting TIM-4 with a monoclonal antibody (mAb) could modify autoimmune responses in NOD mice with new-onset diabetes. Using MHC class I tetramers specific for insulin- and IGRP-derived peptides, we examined β-cell antigen-specific CD8^+^ T cells in lymphoid tissues and the pancreas. We further assessed whether anti-TIM-4 mAb treatment was associated with restoration of euglycemia, preservation of pancreatic islet morphology, and changes in inflammatory and regulatory T-cell phenotypes. These experiments were designed to determine whether modulation of TIM-4-associated immune pathways could alter established diabetogenic T-cell responses in recent-onset autoimmune diabetes.

## 2. Materials and Methods

### 2.1. Mice

Female NOD (NOD/LtJ) mice were purchased from The Jackson Laboratory (Bar Harbor, ME, USA) at 4 weeks of age and maintained under specific pathogen-free conditions. All experiments were performed using female mice. Non-diabetic NOD mice were untreated female NOD mice that remained normoglycemic and had not met the predefined diagnostic criteria for diabetes at the time of tissue collection. These mice were selected as age-matched, contemporaneous controls for the anti-TIM-4-treated and untreated new-onset diabetic NOD mice. All animal experiments were approved by the Kyungpook National University Institutional Animal Care and Use Committee (KNU-2023-0229) and conducted in accordance with institutional guidelines.

### 2.2. Blood Glucose Monitoring and Definition of Diabetes

Non-fasting blood glucose was measured twice weekly using an Accu-Chek glucose monitoring system (Roche, Indianapolis, IN, USA). Non-fasting blood glucose was monitored daily during the period surrounding diabetes onset and longitudinally after anti-TIM-4 mAb treatment. New-onset diabetes was defined as a non-fasting blood glucose level >250 mg/dL on three consecutive days. Restoration of euglycemia following anti-TIM-4 mAb treatment was defined as a random blood glucose level < 200 mg/dL on at least two consecutive measurements.

### 2.3. MHC Class I Tetramer Staining

β-cell antigen-reactive CD8^+^ T cells were identified with MHC class I tetramers generated from biotinylated H-2K^d^/LYLVCGERL (insulin peptide), H-2K^d^/VYLKTNVFL (IGRP_206–214_), and H-2K^d^/KYQAVTTTL (TUM, negative control) monomers provided by the NIH Tetramer Core Facility (Atlanta, GA, USA). Monomers were tetramerized with phycoerythrin (PE)-conjugated streptavidin at a 4:1 monomer-to-streptavidin molar ratio. Single-cell suspensions from spleen, pancreatic lymph nodes, other lymph nodes (inguinal, axillary, and submandibular lymph nodes), and pancreas were stained with allophycocyanin-conjugated anti-CD8 antibody and PE-labeled tetramer and analyzed by flow cytometry.

### 2.4. Generation, Characterization, Production, Purification, and Administration of Anti-TIM-4 mAb

A rat anti-mouse TIM-4 monoclonal antibody, clone 5G3 (rat IgG1), was generated and characterized as previously described [14]. Briefly, a female Lewis rat was immunized with recombinant mouse TIM-4-Ig fusion protein, and lymph-node cells were fused with the established murine myeloma cell line Sp2/0-Ag14, originally described by Shulman et al. [15] and corresponding to ATCC CRL-1581. Hybridoma supernatants were screened by enzyime-linked immunosorbent assay for binding to mouse TIM-4-Ig versus control human cytotoxic T-lymphocyte-associated antigen-4-Ig and subsequently by flow cytometry using cells expressing mouse TIM-4 and control TIM-1 or TIM-3. Clone 5G3 showed specific recognition of TIM-4 without detectable cross-reactivity with the other TIM family members tested [14]. Previous functional characterization demonstrated that anti-TIM-4 monoclonal antibodies, including clone 5G3, partially inhibited TIM-4-dependent T-cell proliferation induced by TIM-4-expressing CHO cells, supporting blocking activity [14]. Because functional antagonism was not independently reassessed in the present study, the antibody is referred to herein as an anti-TIM-4 or TIM-4-targeting monoclonal antibody. For in vivo administration, clone 5G3 hybridoma cells were expanded in five roller bottles containing a total of 5 L DMEM supplemented with 10% IgG-depleted fetal bovine serum. Culture supernatants were collected and passed through a 0.22-μm filter. The antibody was purified using a HiTrap Protein G HP affinity column. After washing with PBS and 0.1 M citric acid (pH 5.3), bound antibody was eluted with 0.1 M glycine (pH 3.5)/0.5 M arginine and immediately neutralized with 1 M Tris (pH 9.0). The antibody was dialyzed overnight against PBS, further buffer-exchanged, and concentrated using an Amicon Ultra centrifugal filter device (Millipore, Billerica, MA, USA). Analysis of the purified preparation demonstrated immunoglobulin heavy- and light-chain bands at approximately 50 and 25 kDa, respectively. (Supplementary Figure S1). New-onset diabetic NOD mice received anti-TIM-4 mAb at 3 mg/kg intraperitoneally on days 0, 2, 4, and 6. Mice were subsequently monitored longitudinally for restoration and maintenance of euglycemia after completion of the four-dose treatment course.

### 2.5. Pancreatic Histology

Animals were anesthetized with pentobarbital (Nembutal; Sigma-Aldrich, St. Louis, MO, USA). Pancreases were excised, weighed, fixed in Bouin’s solution, embedded in paraffin, and sectioned at 5 μm. Sections were immunostained using rabbit anti-insulin antibody (1:200; Millipore, Billerica, MA, USA) with a peroxidase–antiperoxidase method.

### 2.6. Intracellular Cytokine and FoxP3^+^ Staining

For intracellular interferon-γ (IFN-γ) and interleukin-17 (IL-17) staining, lymphocytes isolated from new-onset diabetic NOD mice with or without anti-TIM-4 mAb treatment were stimulated with leukocyte activation cocktail and GolgiPlug (BD Pharmingen, San Diego, CA, USA) for 6 h. Cells were stained with allophycocyanin-conjugated anti-mouse CD4 and/or fluorescein isothiocyanate-conjugated anti-mouse CD8 antibodies and incubated at 4 °C for 30 min in the dark. The cells were then fixed and permeabilized using the Cytofix/Cytoperm kit (BD Pharmingen, San Diego, CA, USA) according to the manufacturer’s instructions and stained with PE-conjugated anti-IFN-γ or anti-IL-17 antibodies. For forkhead box P3 (FoxP3) staining, lymphocytes isolated from new-onset diabetic NOD mice with or without anti-TIM-4 mAb treatment were stained with allophycocyanin-conjugated anti-mouse CD4 and PE-conjugated anti-mouse FoxP3 antibodies (eBioscience, San Diego, CA, USA) without additional stimulation.

### 2.7. Tissue Collection

Anti-TIM-4 mAb treated new-onset diabetic NOD mice that achieved and maintained euglycemia were followed longitudinally and sacrificed approximately 120 days after treatment initiation. At this experimental endpoint, treated mice were sacrificed for immunologic and histologic analyses. Age-matched untreated new-onset diabetic NOD mice and age-matched non-diabetic NOD mice were sacrificed contemporaneously and analyzed in parallel. Because spontaneous diabetes onset occurred at different ages, controls were matched to treated mice by age at the time of tissue collection rather than by a single predefined chronological age.

### 2.8. Statistical Analysis

Data are presented as mean ± standard error of the mean, consistent with the original analysis. The statistical analyses were conducted using a one-way analysis of variance followed by Tukey’s post hoc test performed with GraphPad Prism v.5.01 software (GraphPad Software, La Jolla, CA, USA). *p* value < 0.05 was considered statistically significant.

## 3. Results

### 3.1. Short-Course Anti-TIM-4 mAb Treatment Restores Euglycemia in New-Onset Diabetic NOD Mice

New-onset diabetic NOD mice received anti-TIM-4 mAb (3 mg/kg intraperitoneally every 2 days for four doses). All untreated diabetic NOD mice remained hyperglycemic. In contrast, 8 of 11 anti-TIM-4 mAb-treated mice (72.7%) achieved euglycemia, defined as a random blood glucose level < 200 mg/dL on at least two consecutive measurements. The kinetics of reversal varied among individual responders, with euglycemia being achieved approximately 3 days to 6 weeks after completion of the four-dose treatment course. Once established, glycemic remission was sustained for at least 120 days, despite cessation of anti-TIM-4 treatment. Longitudinal blood glucose profiles of anti-TIM-4-treated and control NOD mice are shown in Supplementary Figure S2.

### 3.2. IGRP_206–214_-Reactive CD8^+^ T Cells Are Enriched in Pancreatic Lymph Nodes and Pancreas of Untreated New-Onset Diabetic NOD Mice Compared to Those of Non-Diabetic NOD Mice

**Figure 1:**
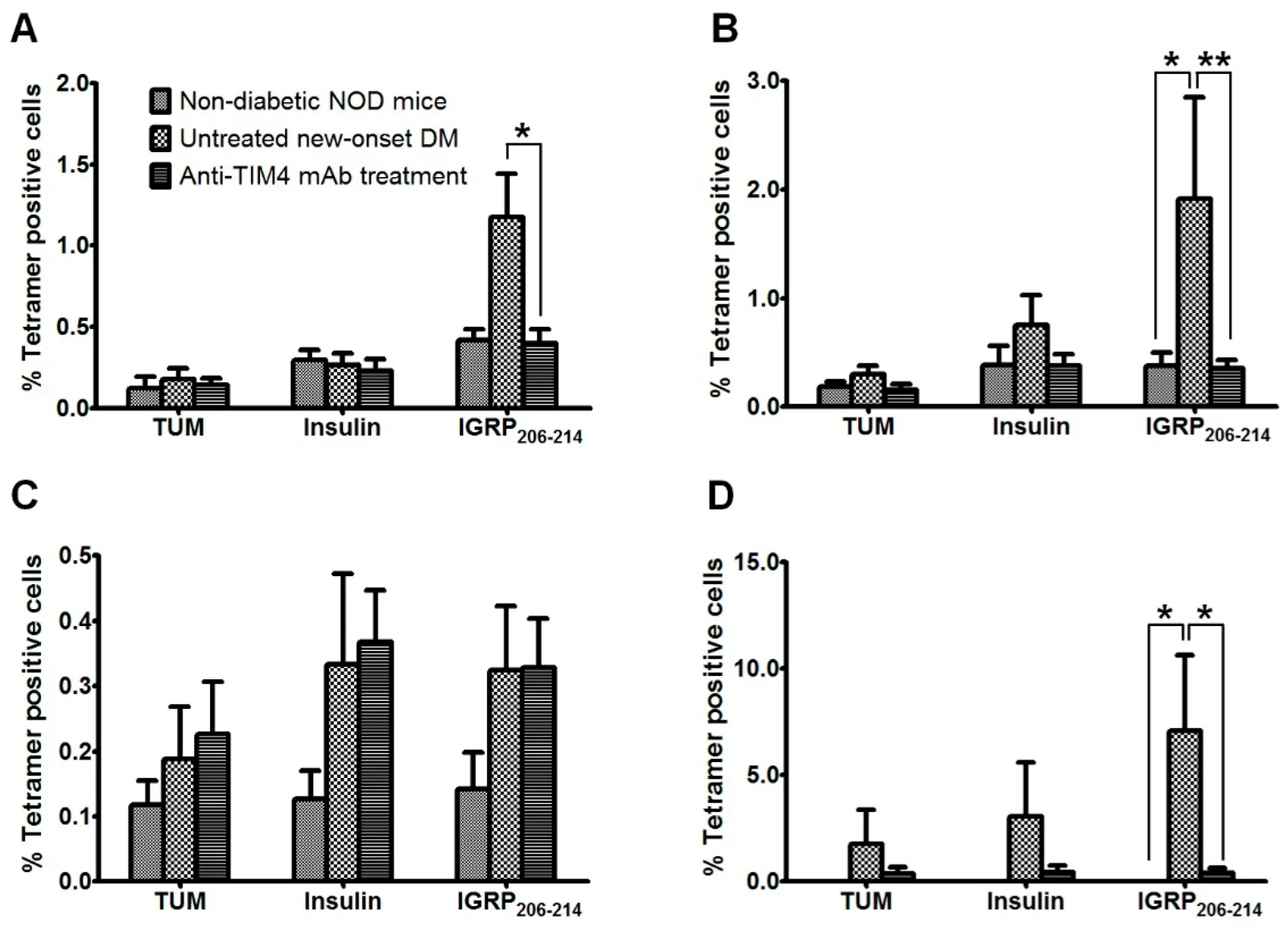
Frequencies of TUM-control, insulin-peptide-reactive, and IGRP_206–214_-reactive MHC class I tetramer-positive CD8^+^ T cells in spleen (**A**), pancreatic lymph nodes (**B**), other lymph nodes (**C**), and pancreas (**D**) of non-diabetic NOD mice (*n* = 4), untreated new-onset diabetic NOD mice (*n* = 6), and anti-TIM-4 mAb-treated new-onset diabetic NOD mice (*n* = 6). Anti-TIM-4 mAb treated mice that had achieved and maintained euglycemia were analyzed approximately 120 days after treatment initiation. Age-matched untreated new-onset diabetic and non-diabetic NOD mice were sacrificed contemporaneously and analyzed in parallel. Non-diabetic NOD mice were female NOD mice that remained normoglycemic and had not met the diagnostic criteria for diabetes at the time of tissue collection. IGRP_206–214_-reactive CD8^+^ T cells differed significantly between non-diabetic and untreated new-onset diabetic NOD mice in pancreatic lymph nodes and pancreas (*p* < 0.05), and between untreated new-onset diabetic NOD mice and anti-TIM-4 mAb-treated new-onset diabetic NOD mice in the spleen and pancreas (*p* < 0.05) and pancreatic lymph nodes (*p* < 0.01). Data are presented as mean ± standard error of the mean. * *p* < 0.05, ** *p* < 0.01.

To determine whether β-cell antigen-reactive CD8^+^ T cells are enriched at the onset of diabetes in NOD mice, we used MHC class I tetramers specific for insulin peptide and IGRP_206–214_. IGRP_206–214_-reactive CD8^+^ T cells were significantly more frequent in pancreatic lymph nodes (1.91 ± 0.94% vs. 0.38 ± 0.12%; *p* < 0.05) and pancreatic infiltrates (7.09 ± 3.53% vs. 0%; *p* < 0.05) of untreated new-onset diabetic NOD mice compared to those of non-diabetic NOD mice. Frequencies of TUM-control and insulin-peptide-reactive CD8^+^ T cells did not differ significantly between these groups (Figure 1).

### 3.3. Anti-TIM-4 mAb Treatment Is Associated with a Marked Reduction in IGRP_206–214_-Reactive CD8^+^ T Cells

**Figure 2:**
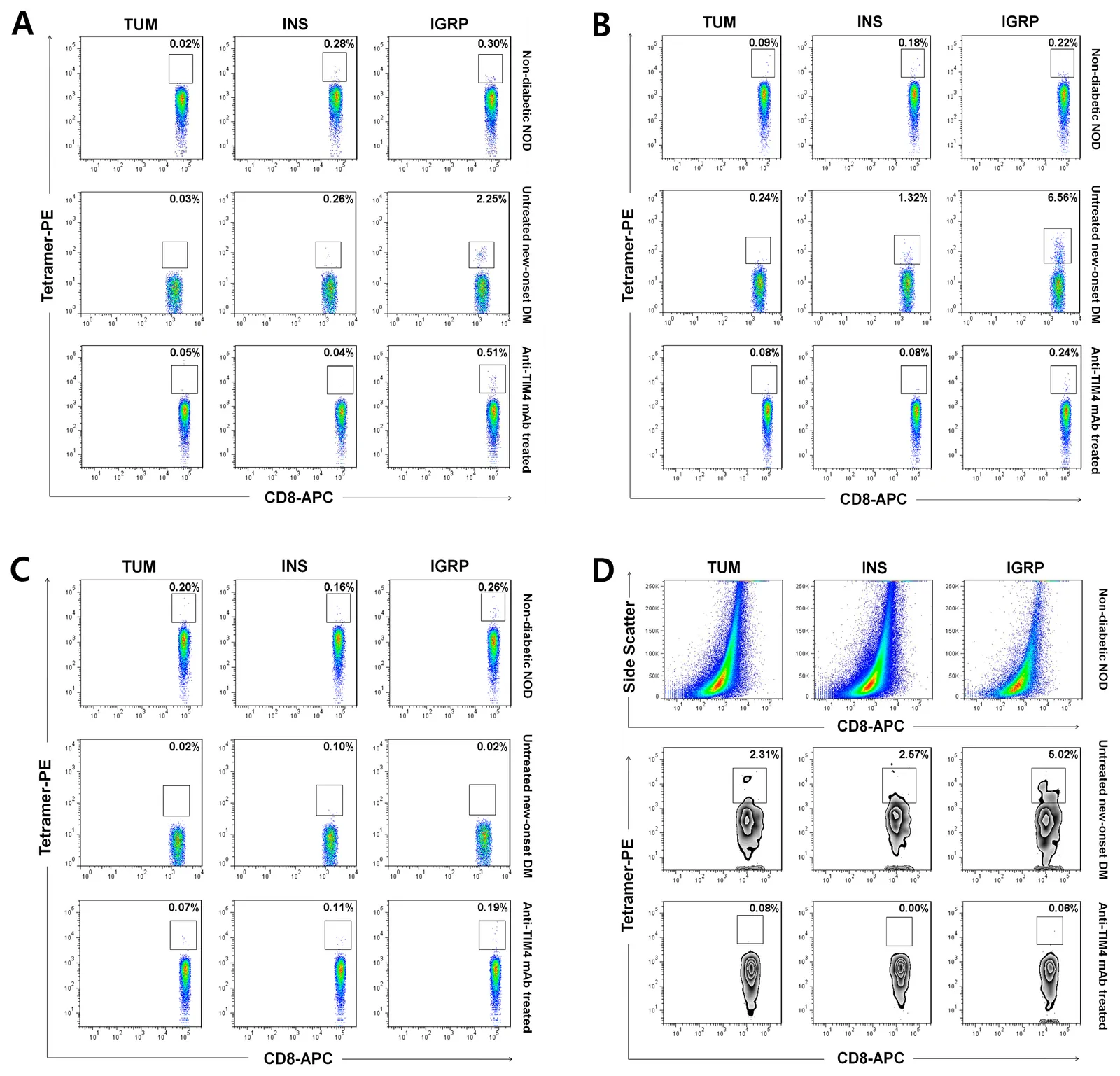
Representative MHC class I tetramer staining patterns in spleen (**A**), pancreatic lymph nodes (**B**), other lymph nodes (**C**), and pancreas (**D**) from non-diabetic NOD mice, untreated new-onset diabetic NOD mice, and anti-TIM-4 mAb-treated new-onset diabetic NOD mice. Samples were obtained approximately 120 days after initiation of anti-TIM-4 mAb treatment from treated mice that had achieved and maintained euglycemia, together with age-matched untreated new-onset diabetic and non-diabetic NOD mice analyzed at the same experimental endpoint. Values within plots indicate the percentage of tetramer-positive cells within the gated CD8^+^ T-cell population. INS, insulin peptide (H-2K^d^/LYLVCGERL) tetramer; IGRP, islet-specific glucose-6-phosphatase-related protein peptide tetramer (H-2K^d^/VYLKTNVFL); TUM, negative-control tetramer (H-2K^d^/KYQAVTTTL).

Compared with untreated new-onset diabetic NOD mice, anti-TIM-4 mAb-treated new-onset diabetic NOD mice had significantly lower frequencies of IGRP_206–214_-reactive CD8^+^ T cells in spleen (0.39 ± 0.09% vs. 1.18 ± 0.26%; *p* < 0.05), pancreatic lymph nodes (0.36 ± 0.08% vs. 1.91 ± 0.94%; *p* < 0.01), and pancreas (0.43 ± 0.23% vs. 7.09 ± 3.53%; *p* < 0.05) (Figure 1). With the exception of other non-pancreatic lymph nodes, frequencies of autoreactive CD8^+^ T cells in anti-TIM-4 mAb-treated new-onset diabetic NOD mice were comparable to those in non-diabetic NOD mice. Representative tetramer staining patterns are shown in Figure 2. CD8^+^ T cells were not detected in the pancreatic samples from the non-diabetic NOD mice analyzed in this experiment.

### 3.4. Anti-TIM-4 mAb Treatment Preserves Insulin-Containing Pancreatic Islets

**Figure 3:**
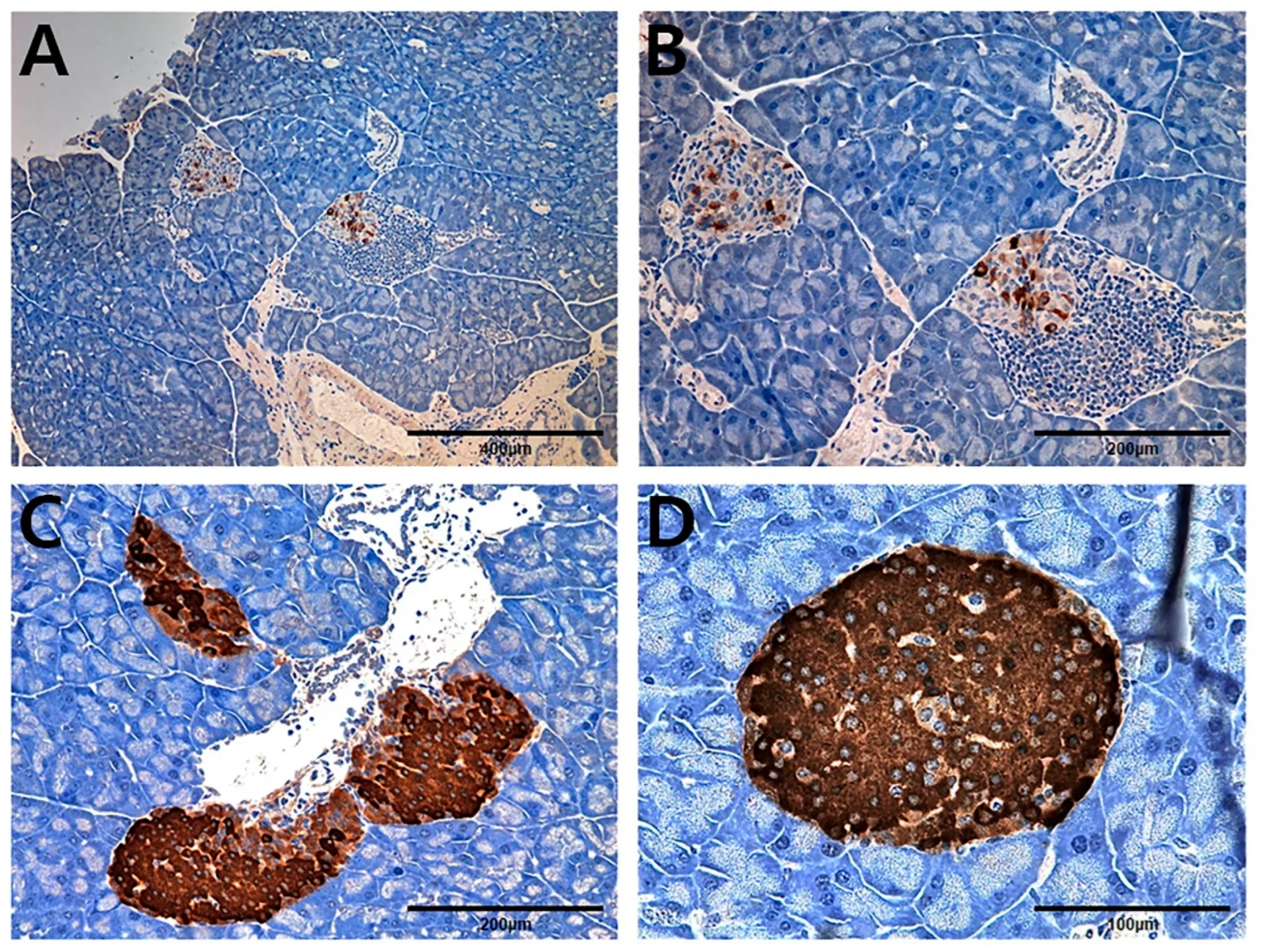
Pancreatic islet histology in untreated (**A**,**B**) and anti-TIM-4 mAb-treated (**C**,**D**) new-onset diabetic NOD mice. Pancreatic tissue from anti-TIM-4 mAb treated mice was obtained approximately 120 days after treatment initiation, after sustained restoration of euglycemia. Age-matched untreated new-onset diabetic NOD mice were sacrificed and examined contemporaneously. In untreated mice, islets were atrophic, with few insulin-positive β-cells ((**A**), ×100), and showed invasive leukocytic infiltration and β-cell degranulation ((**B**), ×200). After anti-TIM-4 mAb treatment and restoration of euglycemia, insulin-containing islets were preserved with markedly less lymphocytic infiltration ((**C**), ×200), and a greater proportion of β-cells appeared granulated ((**D**), ×400). Insulin immunostaining was used in all panels.

In untreated new-onset diabetic NOD mice, most islets were atrophic, contained few insulin-positive β-cells, and showed invasive leukocytic infiltration and β-cell degranulation. In contrast, pancreatic sections from anti-TIM-4 mAb-treated new-onset diabetic NOD mice that returned to euglycemia showed preservation of insulin-containing islets, markedly less peri-islet and intra-islet lymphocytic infiltration, and a greater proportion of granulated β-cells (Figure 3).

### 3.5. Anti-TIM-4 mAb Treatment Is Associated with Changes in Effector Cytokines and FoxP3 Expression in Pancreatic Lymph-Node T Cells

**Figure 4:**
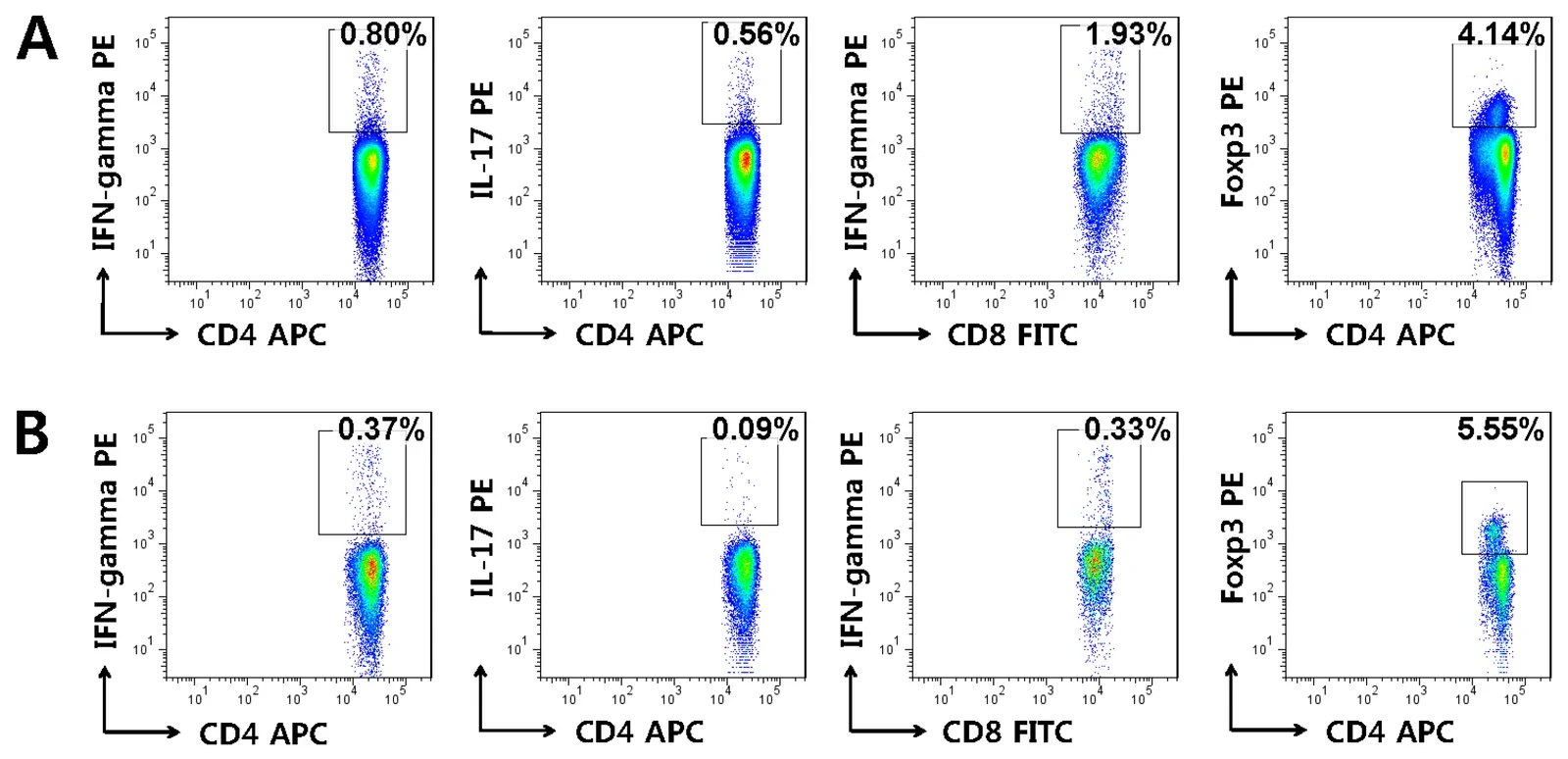
Representative intracellular IFN-γ, IL-17, and FoxP3^+^ staining of lymphocytes isolated from pancreatic lymph nodes of untreated (**A**) and anti-TIM-4 mAb-treated (**B**) new-onset diabetic NOD mice. Samples were collected approximately 120 days after anti-TIM-4 mAb treatment initiation from treated mice that had achieved and maintained euglycemia. Age-matched untreated new-onset diabetic NOD mice were sacrificed contemporaneously and analyzed in parallel. Anti-TIM-4 mAb treatment was associated with lower IFN-γ and IL-17 expression and a higher frequency of FoxP3^+^ CD4^+^ T cells. Values within plots indicate the percentage of cells positive for the indicated marker within the relevant gated T-cell population.

To characterize immune changes associated with treatment, intracellular levels of IFN-γ, IL-17, and FoxP3^+^ were assessed in pancreatic lymph node T cells from untreated and anti-TIM-4 mAb-treated new-onset diabetic NOD mice. Compared with untreated new-onset diabetic mice, anti-TIM-4 mAb-treated mice showed significantly lower IFN-γ expression in CD4^+^ T cells (0.51 ± 0.05% vs. 0.87 ± 0.04%, respectively; *p* < 0.01) and lower IL-17 expression in CD4^+^ T cells (0.27 ± 0.05% vs. 0.57 ± 0.03%; *p* < 0.01). IFN-γ expression in CD8^+^ T cells was also markedly lower in the anti-TIM-4 mAb-treated group than in the untreated group (0.65 ± 0.11% vs. 2.10 ± 0.10%; *p* < 0.01). In contrast, the frequency of FoxP3^+^ CD4^+^ T cells was significantly higher following anti-TIM-4 mAb treatment (5.30 ± 0.14% vs. 4.19 ± 0.17%; *p* < 0.01). Representative intracellular staining patterns are shown in Figure 4. These observations indicate an association between treatment and a shift toward a less inflammatory immune phenotype but do not, by themselves, establish functional regulatory T (Treg)-cell induction.

## 4. Discussion

In this study, a short course of anti-TIM-4 mAb was associated with restoration of euglycemia in 8 of 11 NOD mice with new-onset diabetes and with a marked reduction in IGRP_206–214_-reactive CD8^+^ T cells in pancreatic lymph nodes and pancreatic infiltrates. Histologically, treated mice that became euglycemic showed preservation of insulin-containing islets and less invasive insulitis. These findings suggest that the metabolic response to anti-TIM-4 mAb treatment was accompanied by substantial attenuation of the diabetogenic antigen-specific CD8^+^ T-cell response. The preservation of insulin-containing β-cells is also relevant to the recognized heterogeneity of insulitis and residual β-cell mass during T1D progression [16]. Importantly, recent studies in NOD mice have reinforced the concept that successful reversal of recent-onset diabetes may require simultaneous modulation of pathogenic immunity and preservation of residual β-cell function [5,6].

Autoreactive CD8^+^ T cells are important effectors of β-cell destruction in NOD mice and human T1D [1,2,3,9,10,11]. Recognition of β-cell-derived peptides presented by MHC class I molecules enables antigen-specific CD8^+^ T cells to accumulate within pancreatic islets and exert cytotoxic effector functions [1,2,3]. Inflammatory signals can further enhance β-cell MHC class I expression, thereby increasing their visibility to cytotoxic T cells [17]. Insulin and IGRP are well-characterized targets of diabetogenic CD8^+^ T-cell responses in the NOD model [9,10,11]. In the present study, IGRP_206–214_-reactive CD8^+^ T cells were markedly increased in pancreatic lymph nodes and pancreas at new-onset diabetes, whereas anti-TIM-4 mAb treatment reduced these cells to frequencies approaching those observed in non-diabetic NOD mice. Recent human β-cell studies further show that interferon exposure increases both cell-surface and soluble human leukocyte antigen class I, reinforcing the link between inflammatory signaling and enhanced β-cell antigen visibility to CD8^+^ T cells [18].

The reduction in IGRP_206–214_-specific CD8^+^ T cells in both the pancreatic lymph nodes and pancreas is particularly noteworthy in light of recent insights into the developmental organization of diabetogenic CD8^+^ T-cell responses. Gearty et al. identified a stem-like population of β-cell-specific CD8^+^ T cells within pancreatic draining lymph nodes of NOD mice that self-renews and continuously generates more differentiated autoimmune mediators that migrate to the pancreas and participate in β-cell destruction [3]. These observations suggest that the pancreatic lymph node is not merely a site of initial T-cell priming but may also serve as a reservoir sustaining ongoing autoimmune CD8^+^ T-cell responses. In this context, the marked reduction in IGRP_206–214_-reactive CD8^+^ T cells observed in the pancreatic lymph nodes after anti-TIM-4 mAb treatment could be biologically important, because modulation of this compartment might reduce the continued supply of pathogenic CD8^+^ T cells to pancreatic islets. However, our study did not characterize the differentiation states, clonality, or trafficking of IGRP_206–214_-specific CD8^+^ T cells; therefore, this mechanistic interpretation remains speculative. Recent analyses of human pancreatic lymph nodes similarly demonstrate tissue-restricted immune perturbations, including enrichment of stem-like CD8^+^ T-cell states and altered regulatory T-cell compartments, while multi-omic profiling has identified disease-associated transcriptional and chromatin programs in pancreatic lymphoid tissue [19,20,21].

TIM-4 biology is complex and context-dependent, extending beyond a simple TIM-1/TIM-4 costimulatory model [12,13,14]. TIM-4 is predominantly expressed by macrophages and subsets of APCs and functions as a phosphatidylserine receptor that mediates the recognition and clearance of apoptotic cells via efferocytosis [12,13]. In addition to apoptotic cell clearance, TIM-4 has been implicated in regulating adaptive immune responses and the homeostasis of antigen-specific T-cell populations. TIM-4-expressing phagocytes can recognize phosphatidylserine on activated or apoptotic antigen-specific T cells and participate in their clearance, thereby influencing the magnitude and persistence of antigen-specific immune responses [12,13]. This aspect of TIM-4 biology raises an important mechanistic question regarding our findings. Previous studies in other immune settings have reported that TIM-4-mediated phagocytosis can promote the removal of phosphatidylserine-expressing antigen-specific T cells, and that TIM-4 blockade can, under some circumstances, increase rather than decrease antigen-specific T-cell responses [12,13]. In contrast, anti-TIM-4 mAb treatment in the present study was associated with a profound reduction in IGRP_206–214_-specific CD8^+^ T cells. Thus, our findings cannot be explained simply by blockade of a conventional TIM-4/TIM-1 costimulatory interaction or by inhibition of TIM-4-mediated T-cell clearance. TIM-4 targeting may instead alter antigen presentation, APC activation, autoreactive T-cell survival or trafficking, or the broader inflammatory microenvironment. It is also possible that the consequences of TIM-4 engagement vary with the activation state and anatomical location of TIM-4-expressing APC populations. Defining which TIM-4-expressing cell populations mediate the therapeutic response will require further investigation.

A previous study by Vergani et al. provides an important context for the present findings [22]. Using the anti-TIM-4 mAb RMT4-53, these investigators examined TIM-4 targeting in both islet allotransplantation and spontaneous autoimmune diabetes. In 10-week-old female NOD mice treated before the onset of overt diabetes, TIM-4 targeting did not significantly delay diabetes development and produced only modest Th1-to-Th2 immune skewing. In contrast, RMT4-53 prolonged islet allograft survival in a Th1-dominant transplantation model, whereas it accelerated rejection in a Th2-biased setting; moreover, B-cell depletion abolished the immunologic effects of TIM-4 targeting. These findings indicate that the consequences of TIM-4 modulation are strongly dependent on the immunologic and disease context. Several differences may account for the distinct outcomes between that study and the present experiments. Vergani et al. initiated TIM-4 targeting in prediabetic NOD mice at 10 weeks of age and evaluated prevention of subsequent diabetes, whereas in our study treatment was initiated only after the development of new-onset overt diabetes and was associated with restoration of euglycemia in a subset of mice. The two studies also differed in the anti-TIM-4 antibody used and in the dosing regimen. Furthermore, whereas Vergani et al. emphasized B-cell-dependent modulation of the Th1/Th2 balance, our study identified a marked reduction in IGRP_206–214_-specific CD8^+^ T cells in pancreatic lymph nodes and pancreas together with changes in inflammatory and regulatory T-cell phenotypes. These observations should not be interpreted as establishing distinct mechanisms, because we did not assess B-cell responses or directly identify the TIM-4-expressing cellular population responsible for the therapeutic effect. Rather, the combined findings support a context-dependent role of TIM-4 in regulating anti-islet immunity, potentially influenced by disease stage, antibody characteristics, and the cellular composition of the ongoing immune response.

An additional mechanism that warrants consideration is the effect of TIM-4 targeting on the handling of apoptotic β-cell material by myeloid APCs. TIM-4 recognizes phosphatidylserine exposed on apoptotic cells and dying β-cells represent a potential source of autoantigen during autoimmune diabetes. Recent work has directly demonstrated that intra-islet macrophages in NOD mice engulf apoptotic insulin-positive β-cells and undergo substantial efferocytosis-associated phenotypic remodeling [23]. Interestingly, this process was associated with an anti-inflammatory macrophage phenotype and suppression of autoimmune diabetes, indicating that the consequences of β-cell efferocytosis are complex and potentially tolerogenic. Therefore, altered macrophage-mediated clearance of apoptotic β-cells alone may not fully explain the therapeutic effects observed after TIM-4 targeting. A second, potentially relevant mechanism involves type 1 conventional dendritic cells (cDC1)-mediated cross-presentation of β-cell antigens. cDC1 are specialized for the cross-presentation of cell-associated antigens to CD8^+^ T cells, and selective disruption of WDFY4-dependent cross-presentation in NOD mice markedly impaired autoreactive CD8^+^ T-cell priming and prevented progression to diabetes [24]. TIM-4 has also been shown to mediate the acquisition of dying cell-associated antigens by tissue-resident cDC1; in a tumor model, TIM-4 blockade reduced antigen uptake and cross-presentation to CD8^+^ T cells [25]. These findings raise the hypothesis that anti-TIM-4 treatment in our model may reduce the acquisition and/or cross-presentation of β-cell-derived antigens by cDC1 in pancreatic draining lymph nodes, thereby limiting the maintenance or expansion of IGRP_206–214_-specific CD8^+^ T cells. This mechanism was not directly examined in the present study. Future studies using in vivo labeling of anti-TIM-4-responsive myeloid populations, together with tracking of labeled apoptotic β-cell uptake and antigen cross-presentation, will be required to test this hypothesis. This mechanism is further supported by recent work showing that TYK2 signaling promotes pancreatic lymph-node cross-priming of autoreactive CD8^+^ cytotoxic T cells by resident dendritic cells in NOD mice [26].

Anti-TIM-4 mAb treatment was also associated with reduced IFN-γ and IL-17 expression and a higher frequency of FoxP3^+^ CD4^+^ T cells in pancreatic lymph nodes. These changes are consistent with a shift from a predominantly proinflammatory effector environment toward a more regulated immune state. Regulatory T cells are critical for maintaining peripheral tolerance to β-cell antigens, and abnormalities in Treg stability or function can contribute to the loss of immune tolerance in T1D [27,28]. Nevertheless, the present data demonstrate an association rather than a causal relationship. Intracellular FoxP3^+^ staining alone cannot establish de novo conversion of conventional CD4^+^ T cells into functional Tregs, and our experiments did not determine whether FoxP3^+^ cells were required to restore euglycemia. Functional suppression assays, Treg depletion experiments, and lineage-tracing studies would be necessary to establish such a mechanism. Recent NOD studies further support the therapeutic relevance of this regulatory-effector balance: engineered FoxP3^+^ Tregs redirected toward insulin- or IGRP-specific CD8^+^ T cells suppressed pathogenic responses [29], whereas CD226 co-stimulation blockade enhanced regulatory T-cell activity and diminished effector T-cell function [30].

The restoration of euglycemia after a relatively short immunomodulatory treatment is also relevant to emerging concepts of diabetes reversal in the NOD model. Recent studies demonstrate that established autoimmune diabetes can be reversed in a subset of NOD mice when pathogenic immune responses are sufficiently modified while residual β-cell function is preserved [5,6]. For example, combined β-cell-protective and immunomodulatory therapy has produced durable diabetes reversal associated with reduced severe insulitis, preservation of β-cell mass, and a more favorable regulatory-to-effector T-cell balance [5]. Similarly, short-course anti-CD3 therapy can induce remission in a subset of newly diabetic NOD mice, with responders displaying substantial remodeling of pancreatic T-cell phenotypes [6]. These findings support the concept that reversal of recent-onset diabetes may depend on coordinated changes in both the immune and β-cell compartments rather than on generalized immunosuppression alone. Our observation that anti-TIM-4 mAb treatment was accompanied by reductions in antigen-specific CD8^+^ T cells, changes in inflammatory and regulatory T-cell phenotypes, and preservation of insulin-containing islets is consistent with this model. Consistent with this concept, PD-L1/Gal-9-presenting artificial extracellular vesicles recently reversed new-onset hyperglycemia in NOD mice while reducing pancreatic CD4^+^ and CD8^+^ T-cell infiltration and preserving residual β-cell survival [31].

Several limitations of the present study should be acknowledged. First, the sample size was modest, and the findings require confirmation in larger independent cohorts. Second, the original in vivo experiments did not include an isotype-matched control antibody group. The untreated new-onset diabetic NOD mice therefore controlled for the natural course of disease but did not control for potential nonspecific effects of immunoglobulin administration or Fc receptor engagement. Although the anti-TIM-4 mAb was selected for specific recognition of TIM-4 and treatment was associated with concordant metabolic, immunologic, and histologic changes, the absence of an isotype control limits our ability to attribute all observed effects exclusively to TIM-4-specific targeting. Future studies should include an isotype-matched antibody administered at the same dose and schedule. Third, although anti-TIM-4 mAb treatment was associated with a marked reduction in IGRP_206–214_-reactive CD8^+^ T cells, the underlying mechanism was not directly defined. We did not assess apoptosis, proliferation, migration, or differentiation of these cells, and therefore cannot distinguish between deletion, altered trafficking, impaired expansion, or phenotypic changes as explanations for their reduced frequency. Fourth, the study did not directly identify the TIM-4-expressing APC population responsible for the observed effects. Fifth, the increase in FoxP3^+^ CD4^+^ T cells was not accompanied by functional studies demonstrating enhanced suppressive activity or a requirement for Tregs in diabetes reversal. Finally, contemporary approaches such as single-cell transcriptomic analysis, T-cell receptor clonotype tracking, and spatial characterization of pancreatic immune infiltrates could provide substantially greater mechanistic resolution. These limitations should be considered when interpreting the therapeutic mechanism of anti-TIM-4 mAb treatment.

Despite these limitations, the concordant changes in glycemia, IGRP_206–214_-specific CD8^+^ T-cell frequency, inflammatory and regulatory T-cell phenotypes, and pancreatic islet morphology provide evidence that TIM-4 targeting is associated with substantial remodeling of the autoimmune response in new-onset diabetic NOD mice. In particular, the reduction of IGRP_206–214_-specific CD8^+^ T cells in pancreatic draining lymph nodes and pancreas raises the possibility that TIM-4-associated pathways influence the maintenance and tissue distribution of pathogenic β-cell-specific CD8^+^ T-cell populations.

## 5. Conclusions

In conclusion, short-course anti-TIM-4 mAb treatment was associated with sustained restoration of euglycemia in a substantial proportion of NOD mice with new-onset diabetes and with a marked reduction in diabetogenic IGRP_206–214_-specific CD8^+^ T cells in pancreatic draining lymph nodes and pancreas. These changes were accompanied by preservation of insulin-containing islets, reduced inflammatory T-cell responses, and an increased frequency of FoxP3^+^ CD4^+^ T cells. The persistence of glycemic remission after completion of the short treatment course suggests that TIM-4 targeting may induce durable modulation of the autoimmune response rather than a transient metabolic effect. However, the cellular and molecular mechanisms responsible for these findings remain to be established. In particular, the potential involvement of TIM-4-expressing macrophages and dendritic cells in β-cell antigen uptake, cross-presentation, and maintenance of autoreactive CD8^+^ T-cell responses warrants further investigation. Overall, these findings identify TIM-4-associated immune pathways as potential regulators of β-cell antigen-specific autoimmunity and provide a rationale for further mechanistic evaluation of TIM-4 targeting in type 1 diabetes.

## Data Availability

The data presented in this study are available on request from the corresponding author.

## Supplementary Materials

The following supporting information can be downloaded at: https://www.mdpi.com/article/10.3390/biom16101498/s1, Figure S1: Quality control of purified anti-mouse TIM-4 monoclonal antibody clone 5G3. Analysis of the purified anti-TIM-4 monoclonal antibody preparation demonstrated the expected immunoglobulin heavy-chain (~50 kDa) and light-chain (~25 kDa) bands. Clone 5G3 specificity for TIM-4 was previously characterized by ELISA and flow cytometry using TIM-4 and control TIM-family transfectants as reported in Rodriguez-Manzanet et al. [14]. Figure S2: Longitudinal blood glucose profiles of anti-TIM-4 mAb-treated and control NOD mice. Individual blood glucose measurements are shown longitudinally for an anti-TIM-4 mAb-treated new-onset diabetic NOD mice, an untreated new-onset diabetic NOD mice that subsequently developed spontaneous new-onset diabetes, and a non-diabetic NOD mice. For the anti-TIM-4 mAb-treated mice, day 0 denotes the onset of treatment, and anti-TIM-4 mAb was administered intraperitoneally at 3 mg/kg on days 0, 2, 4, and 6, as indicated by arrows. The anti-TIM-4 mAb-treated responder maintained glycemic control for more than 120 days following treatment. The untreated new-onset diabetic NOD mice were normoglycemic at the beginning of longitudinal monitoring and developed spontaneous diabetes at approximately day 75, after which persistent hyperglycemia was observed. No insulin or other glucose-lowering treatment was administered to the untreated mice. The non-diabetic NOD mouse remained normoglycemic throughout the observation period.

## Abbreviations

The following abbreviations are used in this manuscript:

APCs	antigen-presenting cells
cDC1	Type 1 conventional dendritic cell
FoxP3	forkhead box P3
IFN-γ	interferon-γ
Ig	immunoglobulin
IGRP	islet-specific glucose-6-phosphatase catalytic subunit-related protein
IL-17	interleukin-17
INS	insulin peptide tetramer
mAb	monoclonal antibody
MHC	major histocompatibility complex
NOD	non-obese diabetic
PE	phycoerythrin
T1D	type 1 diabetes
Treg	regulatory T cell
