# Supplementary material for: Anti-TIM-4 Monoclonal Antibody Treatment Reduces Autoreactive CD8+ T Cells and Restores Euglycemia in New-Onset Diabetic NOD Mice
Source: Biomolecules. 2026 Oct 14;16(10):1498. doi: 10.3390/biom16101498 (PMC13702673; doi:10.3390/biom16101498)
Supplement: Supplementary file 1 [file biomolecules-16-01498-s001.zip › biomolecules-4581968-supplementary.pdf]

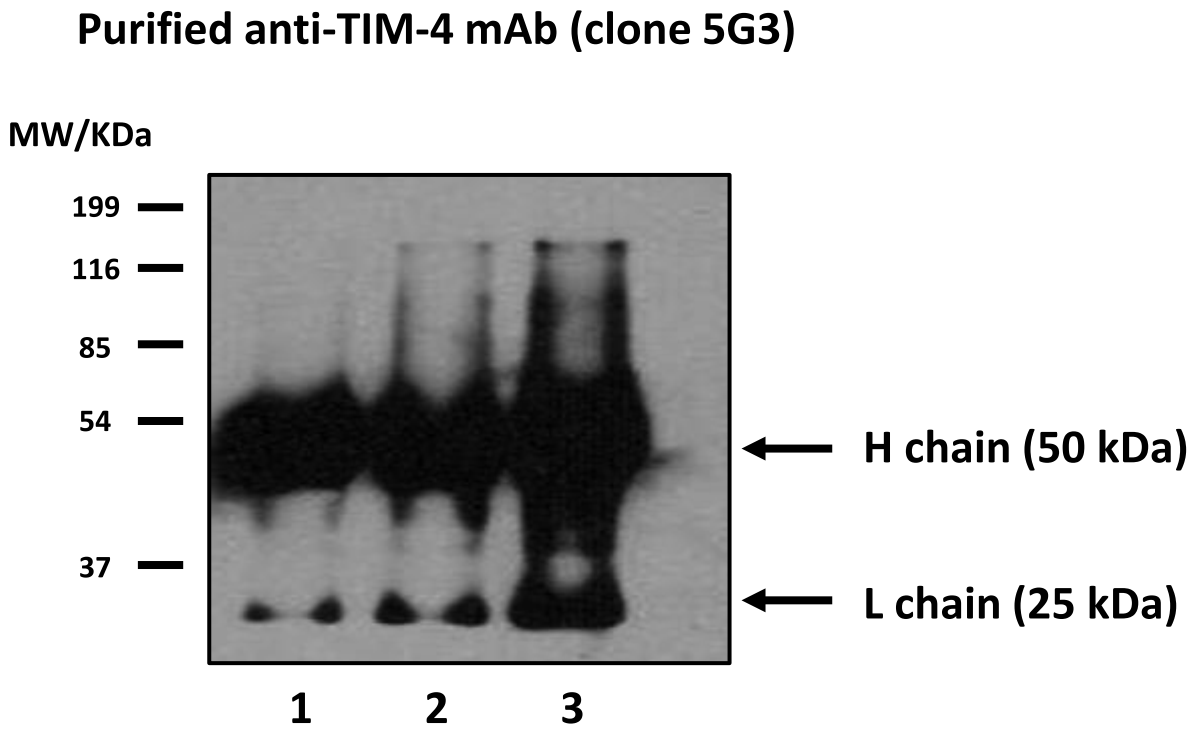

Supplementary Figure S1. Quality control of purified anti-mouse TIM-4 monoclonal antibody clone 5G3. Analysis of the purified anti-TIM-4 monoclonal antibody preparation demonstrated the expected immunoglobulin heavy-chain (~50 kDa) and light-chain (~25 kDa) bands. Clone 5G3 specificity for TIM-4 was previously characterized by ELISA and flow cytometry using TIM-4 and control TIM-family transfectants as reported in Rodriguez-Manzanet et al. [14].

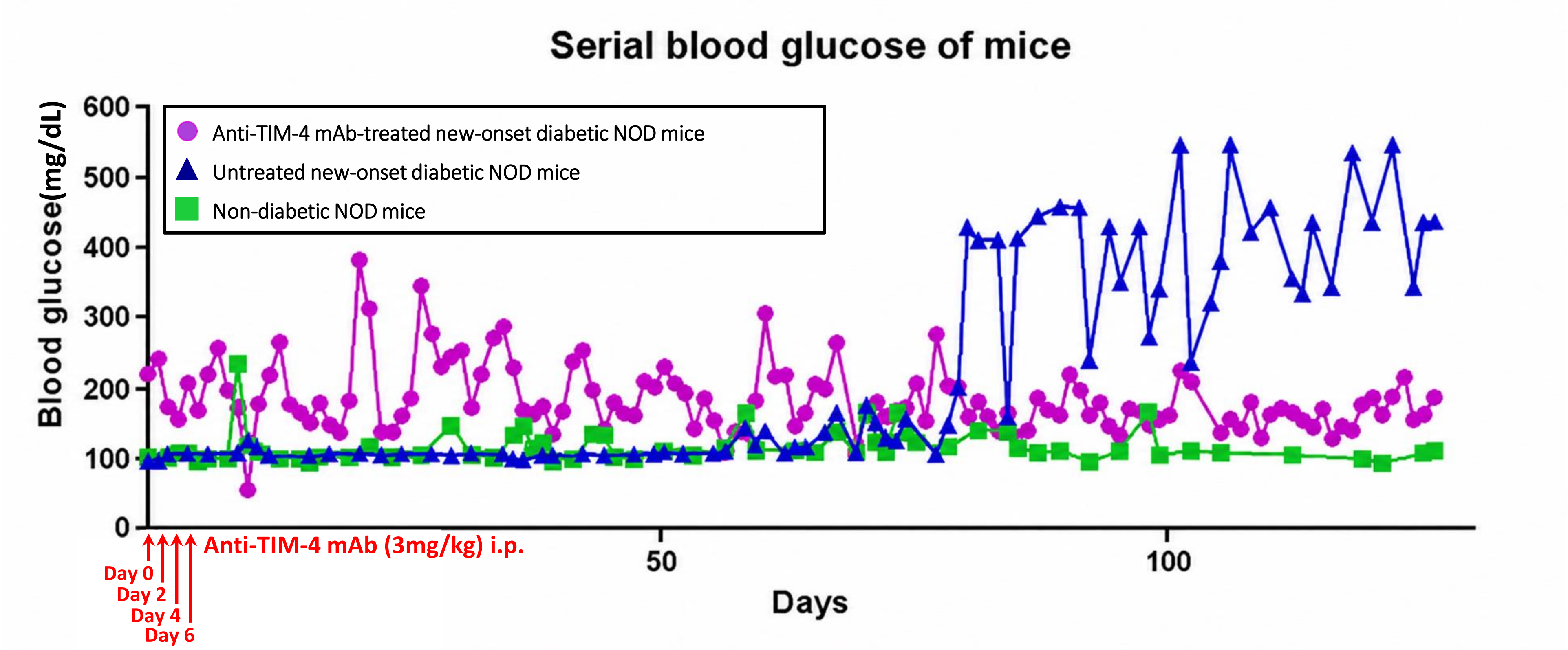

Supplementary Figure S2. Longitudinal blood glucose profiles of anti-TIM-4 mAb-treated and control NOD mice. Individual blood glucose measurements are shown longitudinally for an anti-TIM-4 mAb-treated new-onset diabetic NOD mice, an untreated new-onset diabetic NOD mice that subsequently developed spontaneous new-onset diabetes, and a non-diabetic NOD mice. For the anti-TIM-4 mAb-treated mice, day 0 denotes the onset of treatment, and anti-TIM-4 mAb was administered intraperitoneally at 3 mg/kg on days 0, 2, 4, and 6, as indicated by arrows. The anti-TIM-4 mAb-treated responder maintained glycemic control for more than 120 days following treatment. The untreated new-onset diabetic NOD mice were normoglycemic at the beginning of longitudinal monitoring and developed spontaneous diabetes at approximately day 75, after which persistent hyperglycemia was observed. No insulin or other glucose-lowering treatment was administered to the untreated mice. The non-diabetic NOD mouse remained normoglycemic throughout the observation period.
